# Novel tumor-infiltrating lymphocytes therapy in solid tumors: latest updates from 2025 ASCO annual meeting

**DOI:** 10.1186/s40164-025-00711-x

**Published:** 2025-09-30

**Authors:** Yanan Ma, Xuan Su, Huijing Feng

**Affiliations:** Department of Thoracic Oncology, Cancer Center, Shanxi Bethune Hospital, Shanxi Academy of Medical Sciences, Third Hospital of Shanxi Medical University, Tongji Shanxi Hospital, Taiyuan, 030032 China

**Keywords:** Solid tumors, Adoptive cell therapy (ACT), Tumor-infiltrating lymphocytes (TIL), Clinical study

## Abstract

Tumor-infiltrating lymphocyte (TIL) therapy, a highly promising form of adoptive cell therapy (ACT), has demonstrated success in treating advanced melanoma. Notably, innovative TIL-based monotherapies and combination regimens have provided durable clinical responses and survival benefits for patients with various solid tumors. This article summarizes recent advances in TIL therapy for solid tumors presented at the 2025 ASCO Annual Meeting, highlighting monotherapies such as Lifileucel, LM103, OBX-115, GT101, GT300, GT201, and HS-IT101, as well as combination strategies with the oncolytic adenovirus TILT-123 or pembrolizumab.

## To the editor

Adoptive cell therapy with tumor-infiltrating lymphocytes (TIL-ACT) has demonstrated significant therapeutic potential across a range of solid tumors [1–3]. Recent advancements have established TIL-ACT as a promising treatment for melanoma, leading to its inclusion in the 2024 NCCN guidelines as a preferred second-line regimen for advanced melanoma [4]. However, its efficacy in other solid tumor types remains under active investigation. This report summarizes the latest updates on TIL monotherapies and combination regimens for solid tumors from the 2025 ASCO Annual Meeting, covering efficacy data, technological innovations, and applications across multiple cancer types.

## TIL monotherapies in solid tumors

Lifileucel, an individualized, one-time, tumor-derived autologous T-cell immunotherapy, represents the world’s first FDA-approved TIL therapy for melanoma and has been formally incorporated into the 2024 NCCN Guidelines [4]. Updated 5-year outcomes from the multicenter, multicohort Phase II C-144-01 trial, which enrolled 153 patients with advanced melanoma refractory to PD-1 inhibitors, demonstrated an objective response rate (ORR) of 31.4% (48/153), comprising a 5.9% complete response (CR) rate and a 25.5% partial response (PR) rate. The median duration of response (DOR) was 36.5 months, with 31.3% of responders maintaining their response at 5 years. Median overall survival (OS) was 13.9 months, with a 5-year OS rate of 19.7%. Treatment-emergent adverse events (TEAEs) were predominantly reversible and clinically manageable, with no new safety signals identified during extended follow-up [5].

LM103 is a novel TIL therapy using feeder cell technology to optimize manufacturing efficiency and product quality. In a Phase I trial involving Asian patients with metastatic melanoma that progressed after conventional therapy, patients received an intravenous infusion of autologous LM103 (containing 8.24–19.47 × 10^10^ viable cells) following lymphodepletion and subsequent high-dose IL-2 administration. Among 8 efficacy-evaluable patients, LM103 yielded an ORR of 50% (4/8, all PR). The median progression-free survival (PFS) was not reached, with the longest PFS being 11.4 months. Notably, durable responses were observed in patients with CD8 + T-cell proportions between 60 and 80%. The therapy was well-tolerated with a favorable safety profile [6].

OBX-115 is a next-generation TIL therapy engineered to express membrane-bound IL-15 (mbIL-15) regulated by the small-molecule drug acetazolamide (ACZ), thereby eliminating the need for toxic high-dose IL-2 support post-infusion. In a Phase I multicenter study of 11 patients with advanced melanoma resistant to immune checkpoint inhibitors (ICI), 6 patients received oral ACZ (500 mg/d) followed by OBX-115 infusion (1-100 × 10⁹ cells) after lymphodepletion. An ORR of 67% (4 PR) and a disease control rate (DCR) of 100% were achieved without IL-2. At a median follow-up of 22.3 weeks, the median DOR, PFS, and OS had not been reached. Most TEAEs were reversible and clinically manageable [7].

GT101, another TIL therapy employing a novel manufacturing process to enhance product yield and quality, was initially evaluated in cervical cancer patients. In a multicenter Phase I trial for metastatic cervical cancer refractory to standard treatments, 11 patients received GT101 infusion (dose ≥ 5 × 10⁹ viable cells) after lymphodepletion, followed by high-dose IL-2. The ORR was 45.5% (5/11: 36.4% PR [4/11] and 9.1% CR [1/11]), with a DCR of 90.9% (10/11). The median PFS was 4.83 months, and median OS was not reached. GT101 demonstrated an acceptable safety profile, with the majority of TEAEs being Grade 1–2 in severity [8].

GT300 is the first CRISPR/Cas9 dual knockout anti-exhaustion TIL product to enter pivotal clinical development for solid tumors, designed to enhance TIL function and overcome the immunosuppressive tumor microenvironment (TME). In a Phase I study focusing on advanced, treatment-refractory gynecological cancers, 5 patients received GT300 infusions (1–3 × 10⁹ viable cells) after lymphodepletion, with 4 subsequently receiving IL-2. GT300 demonstrated a favorable clinical profile with ORR 60% (3/5), including CR 40% (2/5) and PR 20% (1/5) and exhibited a favorable safety profile with no long-term safety concerns identified [9].

GT201 is a novel TIL therapy featuring mbIL-15 expression on TILs, aiming to augment immune activation within the TME and eliminate IL-2 toxicity. In a Phase I trial, patients with advanced solid tumors refractory to standard therapies received GT201 infusions (dose ≥ 5 × 10⁹ viable cells) after lymphodepletion, followed by IL-2. Among 9 evaluable patients (with head and neck squamous cell carcinoma [HNSCC], non-small cell lung cancer [NSCLC], melanoma, cervical cancer, and ovarian cancer), the ORR was 55.6% (5/9: 11.1% CR [1/9] and 44.4% PR [4/9]), and the DCR was 77.8% (7/9). Notably, in the HNSCC subgroup, GT201 achieved an ORR of 100% (2/2: 1 PR, 1 CR). All 3 NSCLC patients achieved a DCR of 100% (SD ≥ 24 weeks or PR). No Grade ≥ 3 adverse events were observed [10].

HS-IT101 represents the first TIL therapy developed in China to enter clinical trials for solid tumors. As a novel non-genetically modified TIL product focusing on manufacturing technology advancements, it requires only low-dose lymphodepletion and IL-2 support, significantly reducing safety risks while enhancing cell viability and clinical accessibility. In a Phase I trial for advanced solid tumors, 6 melanoma patients receiving HS-IT101 under this low-intensity regimen achieved an ORR of 50% (16.7% CR [1/6] and 33.3% PR [2/6]) and a DCR of 100%. No serious adverse events (SAEs) were reported [11].

An ongoing Phase 1/2 study is currently evaluating the safety and efficacy of KSQ-004EX, an engineered TIL product with CRISPR/Cas9-mediated dual inactivation of SOCS1 and Regnase-1, in patients with advanced solid tumors [12].

Collectively, these studies indicate that TIL monotherapies can yield substantial clinical efficacy with manageable safety profiles across multiple solid tumor types, establishing them as a valuable treatment option for advanced disease.

## TIL combination regimens in solid tumors

TIL-ACT has been investigated in combination with the oncolytic adenovirus TILT-123 (encoding TNFα and IL-2) in a Phase I trial involving patients with ICI-refractory advanced melanoma. Seventeen patients received intravenous (IV) and intratumoral TILT-123 alongside IV TIL-ACT, without preconditioning chemotherapy or postconditioning IL-2. At the combination stage assessment (Day 78), the ORR was 27%, including one PR lasting 8 months and a durable CR in a case of mucosal melanoma. The DCR by PET criteria was 47%. The median OS (mOS) was 447 days. The combination of TIL-ACT and TILT-123 exhibited a favorable safety profile [13].

In the IOV-COM-202 Phase II open-label study, the administration of Lifileucel plus pembrolizumab as maintenance therapy following standard treatment yielded promising results in anti-PD-1/PD-L1-naïve patients with EGFR wild-type advanced NSCLC, showing an ORR of 35% (7/20). At a median follow-up of 8.2 months, the 6-month PFS rate was 19.7%. No Grade ≥ 3 adverse events were observed [14].

A current Phase Ib study is designed to assess the efficacy and safety of Lifileucel plus adjuvant pembrolizumab in immunotherapy-naïve patients with high-risk, resectable stage IIIb-d melanoma [15].

These results have showed TIL combination regimens as promising strategies with encouraging efficacy and acceptable safety profiles in melanoma and NSCLC.

Despite TIL therapy shows promising prospects, it still faces challenges such as complex manufacturing, limited persistence and immune exhaustion, insufficient clinical evidence beyond melanoma, high costs, and a lack of comparative large-scale randomized study.

## Conclusion

The 2025 ASCO Annual Meeting showcased significant advancements in TIL therapy, as summarized in Tables 1 and 2. Driven by continuous technological innovation, novel TIL therapies characterized by improved safety, enhanced feasibility, and robust efficacy are being developed and are presently under clinical investigation, indicating a promising future for this modality in oncology.

**Table 1:** Outcomes of clinical trials of novel TIL monotherapies in solid tumors from 2025 ASCO annual meeting

Agent	Expansion efficiency	IL-2 dose	IL-15 type	Tumor type	Phase	Accrual	Response	Survival	Major AEs	Abstract Number	References
Lifileucel	Standard	High dose	None	Melanoma	II	153	ORR 31.4%, CR 5.9%, PR 25.5%,mDOR 36.5 m	mOS 13.9 m,5-y OS rate 19.7%	Cytopenia, febrile neutropenia	9515	[5]
LM103	Increased	High dose	None	Melanoma	I	8	ORR50%, PR 50%, mDOR not reached	mPFS not reached, mOS not reached	Emyelosuppression (100%), fever (100%), anemia (100%), and hypotension (100%).	9548	[6]
OBX-115	Increased	None	mbIL-15	Melanoma	I	6	ORR 67%, PR 67%,DCR 100% ,mDOR not reached	mPFS not reached, mOS not reached	Hyponatremia, hypokalemia, CRS event (G2) without IL-6 elevation	9517	[7]
GT101	Increased	High dose	None	Cervical cancer	I	11	ORR 45.5%, PR 36.4%, CR 9.1%, DCR 90.9%, mDOR not reached	mPFS 4.83 m, mOS not reached	Decreased lymphocyte, neutrophil, and white blood cell counts, pyrexia, and tachycardia.	5533	[8]
GT300	Increased	Low dose	None	Solid tumors, focused on gynecological cancers	I	5	ORR 60%, PR 20%, CR40%, mDOR not reached	mPFS not reached, mOS not reached	Fever, rash, dentalulcer, anemia, and decreased platelet count.	5613	[9]
GT201	Increased	Low dose	mbIL-15/IL-15Ra complex	Solid tumors including HNSCC and NSCLC	I	9	ORR 55.6% CR 11.1%,PR 44.4%,DCR 77.8%,mDOR not reached	mPFS not reached, mOS not reached	Decreased lymphocyte, neutrophil, and white blood cell counts, pyrexia, and tachycardia	6038	[10]
HS-IT101	Increased	Low dose	None	Solid tumors	I	6	ORR 50%, CR 16.7%, PR 33.3%, DCR 100%	mPFS not reached, mOS not reached	Fever, fatigue, mild neutropenia and thrombocytop-enia	TPS2674	[11]

*AEs* adverse events, *ORR* objective response rate, *CR* complete response, *PR* partial response, *DCR* disease control rate, *mDOR* median duration of response, *mOS* median overall survival, *mPFS* median progression-free survival, *mbIL-15* membrane-bound IL-15, *CRS* cytokine release syndrome, *HNSCC* head and neck squamous cell carcinoma, *NSCLC* non-small cell lung cancer

**Table 2:** Outcomes of clinical trials of novel TIL combination regimens in solid tumors from 2025 ASCO annual meeting

Agent	Tumor type	Phase	Accrual	Response	Survival	Major AEs	Abstract number	References
TIL+TILT-123	Melanoma	I	17	ORR 27%,DCR 47%	mOS 447 days	N/A	9518	[13]
Lifileucel + pem-brolizumab	NSCLC	II	20	ORR 35%,mDOR not reached	mPFS not reached, mOS not reached	Neutropenia (100%), thrombocytopenia (91%), anemia (84%) pneumonia (9%), colitis (6%), Grade 2 CRS(6%)	TPS8659	[14]

*AEs* adverse events, *ORR* objective response rate, *DCR* disease control rate, *mOS* median overall survival, *mDOR* median duration of response, *mPFS* median progression-free survival, *N/A* not available, *NSCLC* non-small cell lung cancer, *CRS* cytokine release syndrome

## Data Availability

No datasets were generated or analysed during the current study.

## Abbreviations

ACT: Adoptive cell therapy
CR: Complete response
DCR: Disease control rate
DOR: Duration of response
HNSCC: Head and neck squamous cell carcinoma
ICI: Immune checkpoint inhibitors
NSCLC: Non-small-cell lung cancer
ORR: Objective response rate
OS: Overall survival
PFS: Progression-free survival
SAE: Serious adverse events
TEAEs: Treatment-emergent adverse events
TIL: Tumor-infiltrating lymphocytes
TME: Tumor microenvironment
